# The Role of Humeral Neck-Shaft Angle in Reverse Total Shoulder Arthroplasty: 155° versus <155°—A Systematic Review

**DOI:** 10.3390/jcm11133641

**Published:** 2022-06-23

**Authors:** Umile Giuseppe Longo, Lawrence V. Gulotta, Sergio De Salvatore, Alessandra Berton, Ilaria Piergentili, Benedetta Bandini, Alberto Lalli, Vincenzo Denaro

**Affiliations:** 1 Research Unit of Orthopaedic and Trauma Surgery, Fondazione Policlinico Universitario Campus Bio-Medico, Via Alvaro del Portillo, 200, 00128 Roma, Italy; s.desalvatore@unicampus.it (S.D.S.); a.berton@policlinicocampus.it (A.B.); i.piergentili@unicampus.it (I.P.); benedettabandini.000@gmail.com (B.B.); albertolalli30@gmail.com (A.L.); denaro@policlinicocampus.it (V.D.); 2 Research Unit of Orthopaedic and Trauma Surgery, Department of Medicine and Surgery, Università Campus Bio-Medico di Roma, Via Alvaro del Portillo, 21, 00128 Roma, Italy; 3 Centro Integrato di Ricerca (CIR), Campus Bio-Medico University, Via Alvaro del Portillo, 21, 00128 Rome, Italy; 4 Shoulder and Elbow Division of the Sports Medicine Institute, Hospital for Special Surgery, 535 E 70th Street, New York, NY 10021, USA; gulottal@hss.edu

**Keywords:** reverse shoulder arthroplasty, humeral neck-shaft angle, humeral lateralization, center of rotation, outcomes, range of motion, scapular notching

## Abstract

The aim of this study was to have updated scrutiny of the influence of the humeral neck-shaft angle (HNSA) in patients who underwent reverse shoulder arthroplasty (RSA). A PRISMA-guided literature search was conducted from May to September 2021. Clinical outcome scores, functional parameters, and any complications were reviewed. Eleven papers were identified for inclusion in this systematic review. A total of 971 shoulders were evaluated at a minimum-follow up of 12 months, and a maximum of 120 months. The sample size for the “HNSA 155°” group is 449 patients, the “HNSA 145°” group involves 140 patients, and the “HSNA 135°” group comprises 291 patients. The HNSA represents an important variable in choosing the RSA implant design for patients with rotator cuff arthropathy. Positive outcomes are described for all the 155°, 145°, and 135° HSNA groups. Among the different implant designs, the 155° group show a better SST score, but also the highest rate of revisions and scapular notching; the 145° cohort achieve the best values in terms of active forward flexion, abduction, ASES score, and CMS, but also the highest rate of infections; while the 135° design obtains the best results in the external rotation with arm at side, but also the highest rate of fractures. High-quality studies are required to obtain valid results regarding the best prosthesis implant.

## 1. Introduction

Rotator cuff disorders are the most common cause of disability related to the shoulder [1,2]. Currently, cuff tear arthropathy represents a broad spectrum of pathology, in which three critical features are usually present: rotator cuff insufficiency, degenerative changes of the glenohumeral joint, and superior migration of the humeral head [3,4,5,6]. Furthermore, full-thickness rotator cuff tears are present in approximately 25% of individuals in their 60s, and 50% of individuals in their 80s [1].

Reverse shoulder arthroplasty (RSA) was considered a useful solution in these patients, in order to improve their quality of life, restoring a pain-free range of motion (ROM), function, and strength of the shoulder [7,8,9,10,11,12,13,14].

This procedure was described and validated by Paul Grammont in 1985 [15], consisting of an inverted ball and socket joint transplant, where the concavity of the glenoid fossa is replaced with a glenosphere, complementary to a humeral cup [16]. This concept was based on an inversion of the anatomy, enhancing the role of the deltoid muscle in cases of massive rotator cuff tear (MRCT) and cuff tear arthropathy (CTA) [17,18,19].

However, complications rates are reported to range between 39% and 59%, with revision surgery often required [9,18,20,21,22,23,24,25,26]. Thus, some efforts are proposed to reduce the complication rates and improve the ROM, such as inferior glenosphere overhanging, increased lateral offset, and inferior tilting of the baseplate [25]. The lateral offset may be increased either at the humerus, glenoid, or both [27].

The humeral neck-shaft angle (HNSA) is defined as the frontal-plane angle between the humeral proximal articular surface and the intramedullary axis of the humeral shaft, and is likewise highly variable depending on the measurement protocol [28], with reported average measurements between 125° and 150° [29,30]. Grammont revolutionized the design by medializing and distalizing the center of rotation, and utilizing a large convex glenoid surface and concave humeral component with a neck-shaft angle of 155° [17]. However, lateralization of the latter, with the inclination of 135°, is demonstrated to improve the results in ROM [31].

There is still a lack of knowledge about the best location HNSA and, to the best of our knowledge, there are no updated systematic reviews comparing the two prosthesis designs regarding a homogeneous population of patients, in terms of indications for reverse total shoulder arthroplasty (RTSA).

The present systematic review is meant to provide modern knowledge to a less-explored topic in the modern literature regarding the HNSA. The aim is to have updated scrutiny of the possibilities that the various HNSA provide in this type of intervention.

## 2. Materials and Methods

### 2.1. Eligibility Criteria

The research question was formulated using a PICOS-approach: patient (P); intervention (I); comparison (C); outcome (O), and study design (S). The aim of this systematic review was to describe if patients that underwent RTSA (P) with a HNSA of <155° (I) reported better clinical and functional results compared to a HNSA of 155° (C). The outcomes (O) assessed were: active ROM, American shoulders and elbow surgeons (ASES) score, simple shoulder test (SST), absolute Constant–Murley score, visual analogue score (VAS) for pain, scapular notching, complications, and revisions.

To analyze these variables, the review included the following study designs (S): randomized control trials (RCT) and non-randomized controlled studies (NRCT), prospective (PS), retrospective (RS), case-series (CS), case-control (CC), and cohort (C) studies were included.

### 2.2. Inclusion Criteria

Only articles published in English were considered; peer-reviewed articles of each level of evidence according to Oxford classification were screened; studies reporting patients undergoing a primary RTSA were included; and the studies were considered eligible if they specified the humeral neck-shaft angle adopted. In order to be eligible, the indication for RTSA of the patients enrolled in the trials was limited to cuff tear arthropathy, cuff tears, or irreparable cuff tear. In addition, a minimum follow-up of at least 12 months was required.

### 2.3. Exclusion Criteria

Technical notes, letters to editors, instructional courses, or studies including procedures other than reverse shoulder arthroplasty were excluded. Studies that consider revision RTSA, shoulder hemiarthroplasty, and arthroscopic shoulder procedures were discarded. Articles reporting outcomes of patients with indication for surgery as rheumatoid arthritis, acute fracture, post-traumatic fracture sequelae, tumors, or active infection were not considered. In vitro, animal, cadaver, and biomechanical studies were excluded. Studies that do not specify either the prosthesis design or the HNSA, or with missing data, were excluded.

### 2.4. Search

A systematic review was performed using the preferred reporting items for systematic reviews and meta-analyses (PRISMA) guidelines [32]. Medline, EMBASE, Scopus, CINAHL, and CENTRAL bibliographic databases were searched using the following string: (((shoulder) AND ((reverse) OR (total))) AND (((arthroplasty) OR (prosthesis)) OR (replacement))) AND (((humeral) AND (neck)) AND (shaft angle)). The search was performed by two of the authors (B.B. and L.A) from May to September 2021, and articles from inception to 2021 were searched. Keywords were used both isolated and in combination. Additional studies were searched among reference lists of selected papers and systematic reviews.

### 2.5. Data Collection Process

**Figure 1:**
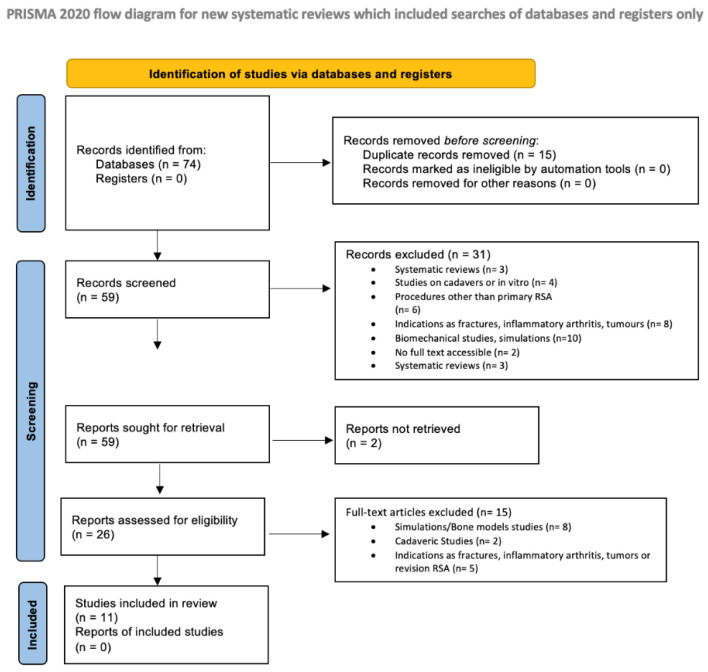
The PRISMA flow-chart of the literature search [32].

Two independent reviewers performed data extraction (B.B. and A.L.), and differences were reconciled by mutual agreement. In case of disagreement for inclusion/exclusion of articles, the consensus of a third reviewer (S.D.S.) was asked. The same authors (B.B. and A.L.) performed the organization and review of the titles to limit the bias. The reviewers used the following research order: titles were screened first, then abstracts, and then full articles. Full text of papers not excluded by title of abstract were evaluated, and eventually selected after a confrontation between the reviewers. The number of articles included or excluded was registered and reported in the PRISMA flowchart (Figure 1). Rules by Moher et al. were followed in designing the PRISMA chart [33].

### 2.6. Data Items

General study characteristics extracted were: author, year of publication, type of study, level of evidence (LOE), sample size, age, gender, and number of shoulders treated (Table 1).

Moreover, prosthesis design, surgical approach, surgical characteristics (considering HNSA, glenosphere size, and glenoid tilt), and follow up were considered (in case of multiple time points, only the last follow-up was reported) (Table 2).

Outcome measures extracted included: absolute Constant–Murley score, ASES score, simple shoulder test (SST) (Table 3), revisions and complications (Table 4); scapular notching was reported following the classification described by Sirveaux [34] (Table 5), active ROMs (reporting forward flexion, abduction, external rotation with the arm at side) (Table 6). For all measurements, the adopted HNSA was specified. Preoperative, postoperative values, and mean standard deviation were reported when present.

**Table 1:** Primary author, year of publication, type of study, level of evidence (LOE), sample size, mean age, gender totals, and number of shoulders treated of the studies included.

Author and Year	Type of Study and Loe	Tot. Sample Size	Sample Size Groups (n)	Shoulders	Tot. Mean Age	Mean Age Groups	Gender	
							M	F
Beltrame et al., 2019 [35]	Prospective study, IV	42	145° (21) 155° (21)	42		145° = 77 155° = 75	12	30
Boutsiadis et al., 2018 [36]	Prognostic study, II	46	155 (I) (13) 155 (III) (11) 145 (II) (10) 145 (IV) (12)	46	77 ± 7.5 (62–90)		9	37
Edwards et al., 2012 [37]	Randomized control trial, treatment study, I	42	155 (T) (20) 155 (NT) (22)	42	69	T = 71.8 ± 8.0 NT = 66.3 ± 9.8	19	23
Franceschetti et al., 2019 [27]	Retrospective cohort study, III	57	145 (29) 145 (BIO-RSA) (30)	59	69.9 ± 8.8	BIORSA = 69.7 ± 9.9 RSA = 70.2 ± 7.8	22	35
Gobezie et al., 2018 [38]	Randomized control trial, I	68	155 (31) 135 (37)		73 (43–94)	155 = 73 135 = 71	155 = 9 135 = 14	155 = 22 135 = 23
Katz et al., 2015 [39]	Retrospective case series study, IV	134	155 (140)	140	72 (52–90)		34	100
Lindbloom et al., 2019 [40]	Retrospective cohort study, III	221	135 (221)	221			88	133
Merolla et al., 2017 [41]	Retrospective cohort study, III	68	155 (36) 145 (38)	74		155 = 75.8 (55–88) 145 = 74.7 (55–91)	155 = 10 145 = 13	155 = 26 145 = 25
Moroder et al., 2016 [26]	Case-control study, III	24	m134 (24)	24	75.6 ± 4.6		7	17
Rhee et al., 2018 [25]	Case-series, IV	138	155 (146)	146	71 ± 5.7		26	112
Streit et al., 2015 [42]	Retrospective case-control study, III	28 (10CG)	155 (9) 135 (9)	28	70.6	155 = 70.9 135 = 70.4	155 = 3 135 = 2	155 = 6 135 = 4

m = mean; CG = control group; T = tilt; NT = no tilt; n = sample size; M = males; F = females; I = medialized COR with neutral glenosphere group; II = lateralized COR with neutral glenosphere group; III = medialized COR with glenoid lateralization (BIO–RSA) group; IV = lateralized COR with glenoid lateralization (BIO–RSA) group.

**Table 2:** Surgical approach, prosthesis design, surgical characteristics, and follow-up of the studies included.

Author and Year	Surgical Approach	Prosthesis Design	Surgical Characteristics			Follow Up (Months)		
			NSA (°)	Glenosphere Size (mm)	Glenoid Tilt	Mean	Max	Min
Beltrame et al., 2019 [35]	Deltopectoral	SMR, Ascend Flex	155, 145			12		
Boutsiadis et al., 2018 [36]	Deltopectoral	Aequalis, Ascend Flex	155, 145	36, 32	Inferior	39 ± 18	84	24
Edwards et al., 2012 [37]	Deltopectoral	Aequalis	155	36	Inferior	21		12
Franceschetti et al., 2019 [27]	Deltopectoral	Aequalis II, Ascend Flex	145					24
Gobezie et al., 2018 [38]	Deltopectoral	Universe Reverse	155, 135	36, 39, 42	Neutral	38	45	29
Katz et al., 2015 [39]	Superior (82.1%), deltopectoral (17.8%)	Arrow	155	36 (83%)	Slightly inferior	45	120	24
Lindbloom et al., 2019 [40]	Deltopectoral	RSP, RSP Monoblock, AltiVate	135					
Merolla et al., 2017 [41]	Deltopectoral	Aequalis II, Ascend Flex	155, 145	36, 42	Centered, inferior	155 = 35.1 145 = 29.1		24
Moroder et al., 2016 [26]	Deltopectoral	TESS	134.4 (116–152)			35	75	24
Rhee et al., 2018 [25]	Deltopectoral	Trabecular metal, Aequalis Biomet comprehensive, Reverse System	155	36	Inferior	20.6	64	12
Streit et al., 2015 [42]	Deltopectoral	Aequalis, Encore	155, 135	36		155 = 9.6 135 = 6.6		

NSA = neck-shaft angle, SMR= Systema Multiplana Randelli, RSP= Reverse Shoulder Prosthesis, TESS= Total Evolutive Shoulder System.

### 2.7. Study Risk of Bias Assessment

Given the designs of the included studies, the risk of bias in non-randomized studies of interventions (ROBINS-I) tool, the risk of bias (RoB 2) tool for randomized trials by Cochrane, and the Joanna Briggs Institute critical appraisal tool for case series were used to assess the quality of each study [44,45,46].

Selected articles were independently rated by each reviewer (B.B, A.L.), and verified by a third one in case of disagreement (S.D.S.).

## 3. Results

### 3.1. Study Selection

The literature search identified 74 articles. No additional studies were found in the grey literature, and no unpublished studies were retrieved. Duplicate removal resulted in the exclusion of 15 studies, leaving 59 articles for screening. A total of 31 articles were excluded based on title and abstract (systematic reviews n = 3; studies on cadavers or in vitro n = 4; procedures other than primary RSA n = 6; indications as fractures, inflammatory arthritis, tumors n = 8; biomechanical studies, simulations n = 10). Moreover, two articles were not retrievable. A total of 26 articles were screened by full text; 15 were excluded (simulations/bone models studies n = 8; cadaveric studies n = 2; indications as fractures, inflammatory arthritis, tumors, or revision RSA n = 5). At the final screening, 11 articles met the selection criteria and were included in the review. The PRISMA flow-chart of the literature search is reported in Figure 1.

### 3.2. Study Characteristics

Two articles [37,38] are randomized controlled studies with level of evidence (LOE) I, one article [35] is a prospective cohort study with LOE II, four articles [27,31,36,40] are retrospective cohort studies with LOE III, two articles [26,42] are retrospective case-control studies with LOE III, and two articles [25,39] are retrospective case series studies with LOE IV. Overall, 971 shoulders are included in the 11 studies. The follow-ups range from a minimum of 12 months to a maximum of 120 months. The sample size for the “HNSA 155°” group is 449 patients, the “HNSA 145°” group involves 140 patients, and the “HSNA 135°” group comprises 291 patients. (Table 1).

Less than two comparative articles included the same data, therefore, it was not possible to perform a meta-analysis.

### 3.3. Quality of Evidence

The RoB 2 tool for RCTs, ROBINS-I tool for NRCTs, and the Joanna Briggs Institute critical appraisal tool for CS were used to assess the methodological quality of each article [44,45,46]. Edwards et al. and Gobezie et al. 2018 [37,38] perform the only RCTs included in this systematic review, and are judged as “low risk of bias”. Out of the nine NRCTs, three are identified as “low risk of bias” studies [26,35,36]; four are identified as “moderate risk of bias” studies [27,31,40,42]; and none result in having serious or critical risk of bias.

CSs are overall of a high quality [25,39].

**Figure 2:**
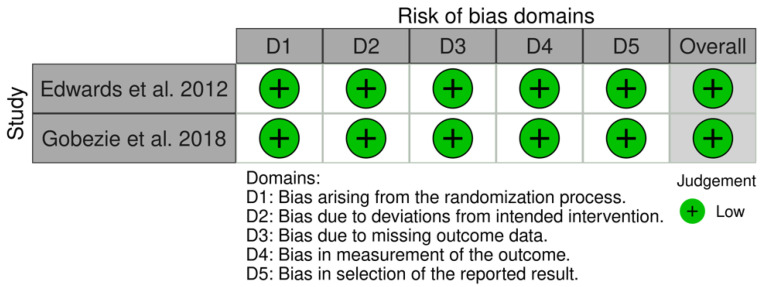
The risk of bias assessments for RCT study [37,38].

**Figure 3:**
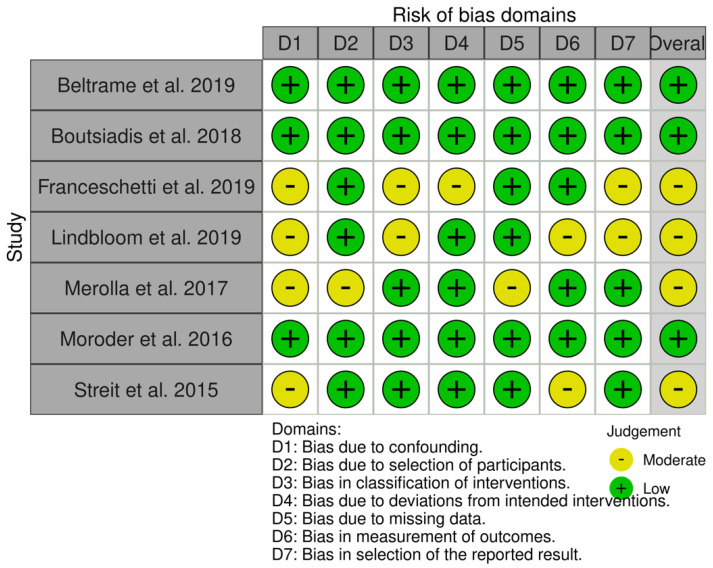
The risk of bias assessments for NRCTs studies [26,27,35,36,40,41,42].

**Figure 4:**
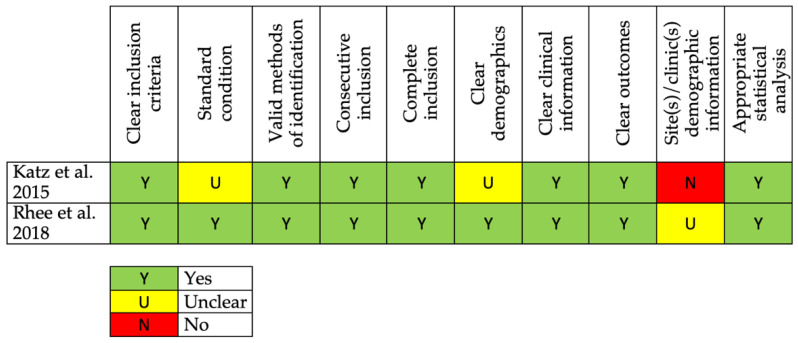
The Joanna Briggs Institute critical appraisal tool for CS studies [25,39].

The risk of bias assessments for RCTs, NRCTs, and CSs are reported in Figure 2, Figure 3 and Figure 4.

### 3.4. Surgical Procedure

Prosthesis design, surgical approach, surgical characteristics (considering humeral neck-shaft angle, glenosphere size, and glenoid tilt), and follow-up are reported in Table 2.

Three studies report the outcomes for 155° HNSA [25,37,39], one study uses a 145° HNSA design [27], and two studies analyze outcomes for 135° HSNA [26,40].

Three studies compare a 155° HNSA to a 145° HNSA [31,35,36], while two studies compare 155° HSNA to a 135° HSNA [38,42].

The patients included in this systematic review were implanted with the following prostheses: SMR [35], Arrow [39], Aequalis [36,37,42], Aequalis II [31,43], RSP Monoblock [40], AltiVate [40], RSP [40], Ascend Flex [27,31,35,36], Encore [42], Aequalis II [27], trabecular metal [25], Biomet Comprehensive [25], Reverse System [25], TESS [26], and Universe Reverse [38].

In all the 11 studies selected, the preferred surgical approach is deltopectoral, with the superior [39] approach as an alternative. The choice of prosthesis design and surgical approach is based on the surgeon’s preference.

The glenosphere sizes reported range from a minimum of 32 mm to 46 mm in diameter for all HNSA groups.

### 3.5. Outcome Score

All the outcomes are reported in Table 3. Seven articles [25,31,35,36,37,39,47] include postoperative CMS, while 6 articles [25,31,35,36,37,39] report both preoperative and postoperative CMS.

Seven papers [25,27,36,38,40,42,47] include the postoperative ASES score, while four papers [25,27,38,40] report both preoperative and postoperative ASES scores.

Five studies [25,36,38,39,40] include the SST score, while three studies [25,38,40] report both the preoperative and postoperative SST score. All three groups (i.e., “155° HNSA”, “145° HNSA”, and “135° HNSA”) show an average improvement in forward flexion, abduction, and external rotation arm at side between preoperative and postoperative follow-up (Table 6).

### 3.6. Complications and Revisions

The most common complications reported are the following: dislocation, fractures, and infection (Table 4).

Three studies report information about dislocations [37,41,48]. In the “155° HNSA” group, three cases of dislocation are shown; in the “145° HNSA” group, there are no reported data about dislocations; and in the “135° HNSA” group, one case of dislocation is shown (Table 5). Considering the three papers with information about dislocations, the rate is 3.8% in the “155° HNSA” group, and 4.2% in the “135° HNSA” group.

Four studies report information about fracture [26,38,39,41]. In the “155° HNSA” group, are seven cases of fractures are shown; in the “145° HNSA” group, there are no reported data about fractures; and in the “135° HNSA” group, three cases of fractures are shown (Table 5). Considering the four papers with information about fractures, the rate is 3.4% in the “155° HNSA” group, and 4.9% in the “135° HNSA” group.

Three studies report information about infection [25,39,41]. In the “155° HNSA” group, six cases of infection are shown; in the “145° HNSA” group, three cases of infection are shown; and in the “135° HNSA” group, there are no reported data about infection. Considering the three papers with information about infections, the rate is 2.1% in the “155° HNSA” group, and 8.3% in the “145° HNSA” group.

Four studies report information about revisions [38,39,40,41]. In the “155° HNSA” group, 16 cases of revisions are shown; in the “145° HNSA” group, two cases of revisions are shown; and in the “135° HNSA” group, four cases of revisions are shown (Table 4). Considering the four papers with information about revisions, the rate is 7.7% in the “155° HNSA” group, 5.6% in the “145° HNSA” group, and 1.6% in the “135° HNSA” group.

### 3.7. Scapular Notching

Seven studies report information about scapular notching [25,27,31,35,37,38,39]. In the “155° HNSA” group, there are 154 cases of scapular notching; in the “145° HNSA” group there are 11 cases; and in the “135° HNSA” group, there are 8 cases (Table 5). Considering the seven papers with information about scapular notching, the rate is 37% in the “155° HNSA” group, 11.6% in the “145° HNSA” group, and 21.6% in the “135° HNSA” group.

### 3.8. Active ROMs

All 11 articles [25,27,31,35,36,37,38,39,40,42,47] include the postoperative forward flexion score, while 8 articles [25,27,31,36,37,38,39,40] report both preoperative and postoperative forward flexion scores.

Seven papers [27,35,36,37,39,40,47] include the postoperative abduction score, while four papers [27,37,39,40] report both preoperative and postoperative abduction scores.

Six studies [27,36,37,38,40,47] include the external rotation arm at side score, while five studies [27,36,37,38,40] report both preoperative and postoperative external rotation arm at side scores. All three groups (i.e., “155° HNSA”, “145° HNSA”, and “135° HNSA”) show an average improvement in forward flexion, abduction, and external rotation arm at side between preoperative and postoperative follow-up (Table 6).

## 4. Discussion

RTSA has become the favored surgical option to reduce pain, improve function, and achieve stability of the joint in rotator cuff arthropathy, severe proximal humeral fractures, and failed anatomic total shoulder arthroplasty [7,8,9,10,11,12,13,14].

Despite this seemingly consistent trend, several studies show that, with the current surgical techniques and implant designs, the procedure is still associated with various problems and complications, such as instability, impingement, infection, component loosening, and periprosthetic fractures [49]. Thus, a total complication rate ranging from 7% to 68% is reported [50,51].

Despite a large amount in the literature on the management of rotator cuff tears, surgical indications remain controversial, and are not standardized [1,52].

The current review reports positive outcomes for all the 155°, 145°, and 135° HNSA groups. Accordingly, in terms of ROM, all three groups show positive outcomes in abduction, external rotation, and forward flexion. Boutsiadis et al. [36] confirm the improvement in external rotation, obtained whether the lateralization was performed at the glenoid (BIO–RSA), or the humeral side (via an on-lay stem). In accordance, Gobezie et al. [38] report improved postoperative values of external rotation and forward flexion, with no difference between humeral inclination of 135° and 155°. Comparing the different implants designs (Table 7), the 145° cohort show greater results in terms of postoperative forward flexion (143° ± 9.8) and external rotation (126.5 ± 12.2), while the 135° cohort show greater results in external rotation (43.2 ± 21.5).

As per the clinical outcomes, the 145° cohort provide better postoperative values in ASES and CMS, while the 155° and 145° both result in the higher postoperative SST values.

Scapular notching rates appear to be higher in the 155° group compared to the other two groups (37%). Scapular notching is less frequent with the use of 135° design compared with the 155° design, but persists at a rate of 21% at a 2 year follow-up [38]. This is coherent with outcomes reported by Oh JH et al. in their cadaveric study, which highlights increased scapular notching in adduction for the 155° cohort, after comparing the 155° design with the 145° model and the 135° one [53]. On the other hand, in the clinical studies conducted by Merolla et al. [31] and Streit et al. [42], no statistically relevant differences are shown in terms of scapular notching.

Revision rates are higher in the 155° HSNA group, in respect of the others (9.4% in contrast to 5.6% of 145°, and 1.6% of 135°). This is confirmed by the studies carried out by Gobezie et al. [38].

Dislocations, fractures, and infections are the complications data included in the qualitative analysis. Dislocations and fractures are reported in the 155° and 135° HSNA groups, with a predominance in the 135° group for both complications (4.2% and 4.9%, respectively). Infections are present in the 155° and 145° HSNA groups, with a prevalence in the 145° cohort (8.3%).

Psychological factors are not assessed in preoperative assessment. However, it is shown that there is a correlation between poor psychological function before surgery and worsening post-surgical outcomes, such as persistence of postoperative pain intensity, and worse levels of function/disability [52,54,55,56].

The recovery of active ROM is reported by Lee et al. as slower in patients with a lateralized humeral stem compared to patients with the standard Grammont procedure, despite lower rates of scapular notching [43]. Lädermann et al. report significant improvements in adduction, external rotation with the arm at side, and extension for varus inclination prostheses (135°–145°). These results are also confirmed by Beltrame et al.; however, the authors only report data at a 6 months follow-up [35,57]. Franceschetti et al. [27] compare outcomes for procedures where humeral lateralization is coupled with glenoid lateralization (BIO–RSA) with procedures applying humeral lateralization alone: external rotation and scapular notching rates improve by humeral lateralization alone, but BIO–RSA presents significantly better results in patients between 50–65 years.

Ferle et al. [50] assess that the 135° neck-shaft angle shows greater stability with the arm in external rotation than 145° and 155° configurations, in their most recent biomechanical study.

The strength of the current systematic review lies in the homogeneity of the patients included in the studies: CTA and rotator cuff tears are the only indications considered for patients included. All the considered studies present with a minimum follow-up of at least 12 months, which allows for an examination of program effects across multiple later life outcomes, as demonstrated by Hill et al. 2016 [58].

No revision surgeries are included, and all sub-populations are stratified accordingly.

### Limitations

This study has some limitations: only two RCTs are present, and not all the considered articles compare all the three parameters in question (HNSA of 155° compared to 145° and 135°). Due to the lack of a valid number of comparative studies, a quantitative analysis was not performed.

The heterogeneous length of follow-up may generate some inconsistency within the outcomes, and the inclusion of only English articles may limit the spectrum current review.

Finally, as observational studies constituted the main source for the analysis, selection bias and confounding due to diverse expectations in RTSA patients should be taken into consideration.

There is still a lack of knowledge about the best value for the HNSA. To the best of the authors’ knowledge, there are no updated systematic reviews comparing the two prosthesis designs regarding a homogeneous population of patients in terms of indications for RTSA.

## 5. Conclusions

The neck-shaft angle seems to represent an important variable in choosing the RSA; however, the lack of comparative data did not allow for meta-analysis on this topic and to obtain significant conclusions.

This systematic review reports the most recent findings on this topic. Positive outcomes are described for the 155°, 145°. and 135° HNSA groups. Among the different implant designs, the 155° group show a better SST score, but also the highest rate of revisions and scapular notching; the 145° cohort achieve the best values in terms of active forward flexion, abduction, ASES score, and CMS, but also the highest rate of infections; the 135° design obtains the best results in the external rotation with arm at side, but also the highest rate of fractures. However, high-quality studies are required to obtain valid results regarding the best prosthesis implant.

## Figures and Tables

**Table 3:** Outcome measures of the studies included (absolute Constant–Murley score, ASES score, and simple shoulder test).

Author and Year	Nsa (°) (n)	Constant–Murley Score		Absolute ASES Score		SST	
		Pre	Post	Pre	Post	Pre	Post
Beltrame et al., 2019 [35]	155 (21)	41	70				
	145 (21)	39	71				
Boutsiadis et al., 2018 [36]	155 (I) (13)	23 ± 3 (12–45)	62 ± 3 (45–71)		75 ± 4 (53–98)		7 ± 0.5 (4–11)
	155 (III) (11)	19 ± 3.5 (2–33)	65 ± 2 (53–77)		77 ± 4 (57–98)		7 ± 0.8 (3–11)
	145 (II) (10)	21 ± 2.5 (8–30)	67 ± 4 (41–86)		79 ± 5 (53–100)		7 ± 1 (2–12)
	145 (IV) (12)	26 ± 1 (16–34)	62 ± 5 (34–87)		72 ± 8 (33–100)		7 ± 1 (1–11)
Edwards et al., 2012 [37]	155 (T) (20)	13.1 ± 9.2	63.6 ± 12.3				
	155 (NT) (22)	15.7 ± 10.8	71.4 ± 14.9				
Franceschetti et al., 2019 [27]	145 (29)			32.7 ± 18.9	80.1 ± 16.7		
	145 (BIO–RSA) (30)			29.4 ± 16.4	77.1 ± 20.9		
Gobezie et al., 2018 [38]	155 (31)			37 ± 18.8	78 ± 15.1	3 ± 2.7	7 ± 2.2
	135 (37)			37 ± 22.6	74 ± 24.6	3 ± 2.8	8 ± 3.0
Katz et al., 2015 [39]	155 (140)	26 (11–53)	64 (26–85)				8.66
Lindbloom et al., 2019 [40]	M = 135 (88)			43 (38–47)	76 (71–81)	3 (2–3)	7 (6–7)
	F = 135 (133)			36 (33–40)	68 (64–72)	2 (1–2)	5 (5–6)
Merolla et al., 2017 [41]	155 (36)	17.9	69.6				
	145 (38)	27	71.2				
Moroder et al., 2016 [26]	m134.4 (24)		65.4 ± 12.9		76.2 ± 10.8		
Rhee et al., 2018 [25]	155 (146)	26 (0–73)	53.9 (23–90)	35.9 (7–72)	67.3 (22–93)	2.6 (0–7)	5.9 (1–10)
Streit et al., 2015 [42]	155 (I) (9)				75.1		
	135 (II) (9)				71		

Pre = pre-operative values; Post = post-operative values; M = males; F = females; T = inferior tilt at the glenoid group; NT = no tilt at the glenoid group; I = medialized COR with neutral glenosphere group; II = lateralized COR with neutral glenosphere group; III = medialized COR with glenoid lateralization (BIO–RSA) group; IV = lateralized COR with glenoid lateralization (BIO–RSA) group; m = mean; n = sample size; ASES= American Shoulder and Elbow Surgeons; SST= Simple Shoulder Test.

**Table 4:** Complications and revisions of the studies included.

Author and Year	Nsa (°) (N)	Complications (N)	Revisions
Beltrame et al., 2019 [35]	155 (21)	/	/
	145 (21)	/	/
Boutsiadis et al., 2018 [36]	155 (I) (13)	/	/
	155 (III) (11)	/	/
	145 (II) (10)	/	/
	145 (IV) (12)	/	/
Edwards et al., 2012 [37]	155 (T) (20)		/
	155 (NT) (22)	(1) dislocation	/
Franceschetti et al., 2019 [27]	145 (29)	0	/
	145 (BIO-RSA) (30)	(1) instability	/
Gobezie et al., 2018 [38]	155 (31)	(3) fractures (1) loosening	4
	135 (37)	(2) fractures (3) loosening	2
Katz et al., 2015 [39]	155 (140)	(4) brachial plexus palsy (1) traumatic fracture of greater tuberosity (1) acromial fracture (6) dissociation of humeral bearing (2) wear of humeral bearing (3) loosening of uncemented humeral (4) glenoid loosening (3) infection (3) stiffness	12 (8.9%)
Lindbloom et al., 2019 [40]	M =135 (88)	(1) glenosphere dissociation (1) instability	2 (0.9%)
	F =135 (133)		
Merolla et al., 2017 [41]	155 (36)	(2) dislocation	0
	145 (36)	(2) scapular spine fracture (1) acromial fracture (3) infection (1) instability	2
Moroder et al., 2016 [26]	134.4 (24)	(1) dislocation (1) acromial spine fracture (1) symptomatic mesacromion (3) stiffness (2) hematomas (1) transient paresthesia (1) inlay snapping	3
Rhee et al., 2018 [25]	155 (146)	(3) infection (7) neurologic complications	/
Streit et al., 2015 [42]	155 (I) (9)	/	/
	135 (II) (9)	/	/

M = males; F = females; T = inferior tilt at the glenoid group; NT = no tilt at the glenoid group; I = medialized COR with neutral glenosphere group; II = lateralized COR with neutral glenosphere group; III = medialized COR with glenoid lateralization (BIO–RSA) group; IV = lateralized COR with glenoid lateralization (BIO–RSA) group.

**Table 5:** Scapular notching of the studies included.

Author and Year	Nsa° (n)	Scapular Notching		Grades of Notching (% or n)			
		N	%	Grade I	Grade II	Grade III	Grade IV
Beltrame et al., 2019 [35]	155 (21)	3	24	3	0	0	0
	145 (21)						
Boutsiadis et al., 2018 [36]	I 155 (13)						
	III 155 (11)						
	II 145 (10)						
	IV 145 (12)						
Edwards et al., 2012 [37]	T 155 (20)	15		5	8	2	
	NT 155 (22)	19		8	10	1	
Franceschetti et al., 2019 [27]	145 (BIO-RSA) (30)	4	13.3	4			
	145 (29)	5	17.2	4	1		
Gobezie et al., 2018 [38]	155 (31)	18	58	5	10	1	2
	135 (37)	8	21	3	3	1	1
Katz et al., 2015 [39]	155 (140)	41	29	20	18	3	0
Lindbloom et al., 2019 [40]	M = 135 (88)						
	F = 135 (133)						
Merolla et al., 2017 [41]	155 (36)	14	39.0	11	1	0	0
	145 (38)	2	5	2	0	0	0
Moroder et al., 2016 [26]	m134.4 (24)			2			
Rhee et al., 2018 [25]	155 (146)	44	30	37	7		
Streit et al., 2015 [42]	155 (9)						
	135 (9)						

T = inferior tilt at the glenoid group; NT = no tilt at the glenoid group; I = medialized COR with neutral glenosphere group; II = lateralized COR with neutral glenosphere group; III = medialized COR with glenoid lateralization (BIO–RSA) group; IV = lateralized COR with glenoid lateralization (BIO–RSA) group.

**Table 6:** Active ROMs (forward flexion, abduction, and external rotation with the arm at side) of the studies included.

Author and Year	Nsa° (n)	ROM					
		Forward Flexion		Abduction		External Rotation Arm at the Side (°)	
		Pre	Post	Pre	Post	Pre	Post
Beltrame et al., 2019 [35]	155 (21)		153		142		−42
	145 (21)		158		144		−37
Boutsiadis et al., 2018 [36]	I 155 (13)	63 ± 21 (10–100)	148 ± 7 (100–170)		134 ± 8.5 (90–170)	14 ± 20 (−30–50)	14 ± 13 (−10–35)
	III 155 (11)	74 ± 35 (10–120)	158 ± 4 (130–175)		145 ± 7 (100–170)	5 ± 20 (−30–40)	24 ± 12 (0–40)
	II 145 (10)	53 ± 22 (30–90)	149 ± 8 (90–175)		134 ± 9 (80–175)	8 ± 21 (−30–20)	31 ± 13 (15–60)
	IV 145 (12)	80 ± 35 (0–120)	152 ± 8 (80–180)		129 ± 11 (80–170)	14 ± 20 (−30–40)	30 ± 16 (0–50)
Edwards et al., 2012 [37]	T (20)	51.6 ± 49.1	156.6 ± 21.2	49.8 ± 49	155.9 ± 21.0	0.7 ± 1.8	8.3 ± 2.6
	NT (22)	36.0 ± 45.6	148.0 ± 19.4	32.3 ± 37.4	141.8 ± 27.3	0.3 ± 1.3	7.4 ± 1.8
Franceschetti et al., 2019 [27]	145 (BIO-RSA) (30)	78 ± 31	136 ± 21	67 ± 28	118 ± 19	15 ± 11	32 ± 20
	145 (29)	81 ± 29	135 ± 25	65 ± 29	119 ± 26	16 ± 11	40 ± 18
Gobezie et al., 2018 [38]	155 (31)	76 ± 50	135 ± 17			29 ± 15	30 ± 14
	135 (37)	78 ± 47	132 ± 19			28 ± 14	29 ± 10
Katz et al., 2015 [39]	155 (140)	73	132	61	108	20	29
Lee et al., 2021 [43]	155 (43)		130 ± 16		127 ± 14		48 ± 14
	145 (71)		132 ± 16		125 ± 16		48 ± 12
Lindbloom et al., 2019 [40]	M =135 (88)	81 (72–90)	151 (142–159)	75 (68–82)	136 (126–146)	32 (24–39)	55 (46–64)
	F =135 (133)	70 (63–78)	136 (128–144)	66 (59–73)	121 (113–130)	26 (19–33)	46 (38–54)
Merolla et al., 2017 [41]	155 (36)	65	142			15	30
	145 (38)	83	142			0	32
Moroder et al., 2016 [26]	m134.4 (24)		7.8 ± 1.9		6.9 ± 2.0		6.6 ± 2.6
Rhee et al., 2018 [25]	155 (146)	96.4	138.4			30.6	48.9
Streit et al., 2015 [42]	155 (9)		143.9				
	135 (9)		115.6				
Teissier et al., 2015 [18]	m154 (91)	96	143	89	138	47	68

m = mean; Pre = pre-operative values; Post = post-operative values; T = inferior tilt at the glenoid group; NT = no tilt at the glenoid group; I = medialized COR with neutral glenosphere group; II = lateralized COR with neutral glenosphere group; III = medialized COR with glenoid lateralization (BIO–RSA) group; IV = lateralized COR with glenoid lateralization (BIO–RSA) group; ROM = range of motion; n = sample size.

**Table 7:** Comparison between different implant subtypes.

	ACTIVE ROM (°)			SCAPULAR NOTCHING	ASES	CMS	SST	REVISIONS	COMPLICATIONS		
	FF	ABD	ER						DISLOCATIONS	FRACTURES	**INFECTIONS**
155°	Pre: 77.4 ± 17.2 Post: 139.3 ± 8.3	Pre: 56.3 ± 14.4 Post: 121.9 ± 18	Pre: 21.4 ± 11.5 Post: 31.3 ± 29.1	154 (37%)	Pre: 36.1 ± 0.8 Post: 70.2 ± 4.7	Pre: 24.6 ± 9.3 Post: 61.5 ± 6	Pre: 2.7 ± 0.3 Post: 7.2 ± 1.1	16 (9.4%)	3 (3.8%)	7 (3.4%)	6 (2.1%)
145°	Pre: 78.3 ± 6.8 Post: 143 ± 9.8	Pre: 66 ± 1.4 Post: 126.5 ± 12.2	Pre: 14.4 ± 7.3 Post: 27.1 ± 31.8	11 (11.6%)	Pre: 31 ± 2.3 Post: 77.7 ± 2.5	Pre: 29.3 ± 8.1 Post: 69.2 ± 3.9	Pre: NA Post: 7	2 (5.6%)	0	0	3 (8.3%)
135°	Pre: 74.9 ± 2.6 Post: 128.8 ± 62	Pre: 69.6 Post: 115.2 ± 84.9	Pre: 28.3 ± 0.3 Post: 43.2 ± 21.5	8 (21.6%)	Pre: 38.5 ± 1.3 Post: 72 ± 2.5	Pre: NA Post: 65.4	Pre: 2.5 ± 0.4 Post: 6.1 ± 1.6	4 (1.6%)	1 (4.2%)	3 (4.9%)	0

Pre = preoperative values; Post = postoperative values, ROM = range of motion, FF = forward flexion, ABD = abduction, ER = external rotation, ASES = American shoulder and elbow surgeons score, CMS = Constant–Murley score, SST = simple shoulder test.

## Data Availability

The datasets analyzed during the current study are not public, but are available from the corresponding author on reasonable request.
